# Post Genome-Wide Association Studies of Novel Genes Associated with Type 2 Diabetes Show Gene-Gene Interaction and High Predictive Value

**DOI:** 10.1371/journal.pone.0002031

**Published:** 2008-05-07

**Authors:** Stéphane Cauchi, David Meyre, Emmanuelle Durand, Christine Proença, Michel Marre, Samy Hadjadj, Hélène Choquet, Franck De Graeve, Stefan Gaget, Frederic Allegaert, Jérôme Delplanque, Marshall Alan Permutt, Jon Wasson, Ilana Blech, Guillaume Charpentier, Beverley Balkau, Anne-Claire Vergnaud, Sébastien Czernichow, Wolfgang Patsch, Mohamed Chikri, Benjamin Glaser, Robert Sladek, Philippe Froguel

**Affiliations:** 1 CNRS 8090-Institute of Biology, Pasteur Institute, Lille, France; 2 INSERM U695, Paris, France; 3 René Diderot-Paris 7 University, Paris, France; 4 Department of Endocrinology-Diabetology and Nutrition, Bichat Claude Bernard Hospital, Paris, France; 5 CHU de Poitiers, Endocrinologie Diabetologie, CIC INSERM 0801, INSERM U927, Université de Poitiers, UFR Médecine Pharmacie, Poitiers, France; 6 Washington University School of Medicine, St. Louis, Missouri, United States of America; 7 Endocrinology and Metabolism Service, Department of Internal Medicine, Hadassah-Hebrew University Medical Center, Jerusalem, Israel; 8 Endocrinology-Diabetology Unit, Corbeil-Essonnes Hospital, Corbeil-Essonnes, France; 9 INSERM U780-IFR69, Villejuif, France; 10 University of Paris-Sud, Paris, France; 11 UMR U557 INSERM; U1125 INRA; CNAM; PARIS 13-CRNH-IdF, Bobigny, France; 12 Hôpital Avicenne, Département de Santé Publique, Assistance Publique-Hôpitaux de Paris (AP-HP), Bobigny, France,; 13 Department of Laboratory Medicine, Paracelsus Medical University and Landeskrankenhaus Salzburg, Salzburg, Austria; 14 Laboratory of Biochemistry, Faculty of Medicine and Pharmacy of Fez, Route Sidi Harazem, Fez, Morocco; 15 Department of Human Genetics, Faculty of Medicine, McGill University, Montreal, Canada; 16 Department of Medicine, Faculty of Medicine, McGill University, Montreal, Canada; 17 Department of Pediatrics, Faculty of Medicine, McGill University, Montreal, Canada; 18 Genomic Medicine, Hammersmith Hospital, Imperial College London, London, United Kingdom; Canadian Agency for Drugs and Technologies in Health, Canada

## Abstract

**Background:**

Recently, several Genome Wide Association (GWA) studies in populations of European descent have identified and validated novel single nucleotide polymorphisms (SNPs), highly associated with type 2 diabetes (T2D). Our aims were to validate these markers in other European and non-European populations, then to assess their combined effect in a large French study comparing T2D and normal glucose tolerant (NGT) individuals.

**Methodology/Principal Findings:**

In the same French population analyzed in our previous GWA study (3,295 T2D and 3,595 NGT), strong associations with T2D were found for *CDKAL1* (OR_rs7756992_ = 1.30[1.19–1.42], *P* = 2.3×10^−9^), *CDKN2A/2B* (OR_rs10811661_ = 0.74[0.66–0.82], *P* = 3.5×10^−8^) and more modestly for *IGFBP2* (OR_rs1470579_ = 1.17[1.07–1.27], *P* = 0.0003) SNPs. These results were replicated in both Israeli Ashkenazi (577 T2D and 552 NGT) and Austrian (504 T2D and 753 NGT) populations (except for *CDKAL1*) but not in the Moroccan population (521 T2D and 423 NGT). In the overall group of French subjects (4,232 T2D and 4,595 NGT), *IGFBP2* and *CXCR4* synergistically interacted with (*LOC38776*, *SLC30A8*, *HHEX*) and (*NGN3*, *CDKN2A/2B*), respectively, encoding for proteins presumably regulating pancreatic endocrine cell development and function. The T2D risk increased strongly when risk alleles, including the previously discovered T2D-associated *TCF7L2* rs7903146 SNP, were combined (8.68-fold for the 14% of French individuals carrying 18 to 30 risk alleles with an allelic OR of 1.24). With an area under the ROC curve of 0.86, only 15 novel loci were necessary to discriminate French individuals susceptible to develop T2D.

**Conclusions/Significance:**

In addition to *TCF7L2, SLC30A8* and *HHEX*, initially identified by the French GWA scan, *CDKAL1, IGFBP2* and *CDKN2A/2B* strongly associate with T2D in French individuals, and mostly in populations of Central European descent but not in Moroccan subjects. Genes expressed in the pancreas interact together and their combined effect dramatically increases the risk for T2D, opening avenues for the development of genetic prediction tests.

## Introduction

Recently, genome-wide association (GWA) studies have revealed new single nucleotide polymorphisms (SNPs) that are strongly associated with type 2 diabetes (T2D) [1]–[5]. In our French study, we showed that the well known rs7903146 *TCF7L2* polymorphism ranked first for its effect on T2D prevalence followed by four new risk loci: *SLC30A8*, *HHEX*, *LOC387761* and *EXT2* [1]. Subsequently, GWA studies in Finnish, English, Icelandic and Danish populations emphasized the role of *CDKAL1*, *CDKN2A/2B* and *IGFBP2* on T2D and confirmed the effect of *TCF7L2*, *SLC30A8* and *HHEX* [2]–[5]. Additional SNPs, located in *MMP26*, *LDLR*, *KCTD12*, *CAMTA1*, *NGN3*, *CXCR4*, *LOC646279*, were also among the 15 first signals in the joint stage I and fast track stage II analyses of the French study [1], but their current status is uncertain as they did not rank high in the other GWA scans [2]–[5]. Although the other loci listed above are likely to be true T2D markers, the actual impact of these genetic variants in other European and non-European populations as well as their cumulative effects and potential interactions remain to be determined.

In the present study, we first investigated the association of *CDKAL1*, *CDKN2A/2B* and *IGFBP2* SNPs with T2D in the French population that we analyzed in our GWA study followed by a fast track replication [3,295 T2D and 3,595 normal glucose tolerant (NGT)] [1]. Then, 22 SNPs in 14 loci previously associated with T2D in GWA studies were selected and analyzed in four additional independent populations of European [French (937 T2D and 1,000 NGT), Austrian (504 T2D and 753 NGT), Israeli Ashkenazi (577 T2D and 552 NGT)] and Moroccan Arabic (521 T2D and 423 NGT) origin. Finally, in the overall group of French individuals (4,232 T2D and 4,595 NGT), we assessed possible gene-gene interactions and determined the cumulative genetic risk of carrying risk alleles on T2D prevalence.

## Results

Clinical characteristics of each population are reported in Table S1. Minimum detectable effect size with a statistical power of 80% was calculated for each SNP in all studied samples and linkage disequilibrium was assessed between genetic variants located in the same locus (Table S2).

### Association studies in our original set of French individuals

We first studied whether 3 risk loci (7 SNPs) identified in Finnish, English, and Icelandic-Danish populations conferred risk in our original French population (3,295 T2D and 3,595 NGT). Polymorphisms of *CDKAL1*, *CDKN2A/2B* and *IGFBP2* were all associated with T2D (OR_rs7756992_ = 1.30 [1.19–1.42], *P* = 2.3×10^−9^; OR_rs10811661_ = 0.74 [0.66–0.82], *P* = 3.5×10^−8^; and OR_rs1470579_ = 1.17 [1.07–1.27], *P* = 0.0003), respectively) (Table 1). In this population and for each SNP, the best mode of inheritance (*P*_max_) was found to be multiplicative.

**Table 1:** Genotypic distribution by number of individuals (%) of the 7 SNPs studied in the first set of French population

Whole-genome study	Gene	rs ID	Genotype 1-1		Genotype 1-2		Genotype 2-2		OR	*P*
			NGT	T2D	NGT	T2D	NGT	T2D	(95% CI)	
**Scott ** [**2**]	*CDKN2A/2B*	rs10811661	2,302 (65)	2,230 (71.3)	1,107 (31.2)	820 (26.2)	134 (3.8)	76 (2.4)	**0.74 (0.66–0.82)**	**3.5×10^−8^**
	*CDKN2A/2B*	rs564398	1,343 (37.8)	1,323 (42.1)	1,659 (46.7)	1,400 (44.6)	548 (15.4)	418 (13.3)	**0.84 (0.77–0.91)**	**2.2×10^−5^**
**Zeggini ** [**3**]	*CDKAL1*	rs7754840	1,690 (47.5)	1,304 (41.5)	1,523 (42.8)	1,439 (45.8)	345 (9.7)	399 (12.7)	**1.23 (1.13–1.33)**	**1.6×10^−6^**
	*CDKAL1*	rs7756992	1,888 (53.3)	1,436 (45.7)	1,394 (39.4)	1,373 (43.7)	258 (7.3)	330 (10.5)	**1.30 (1.19–1.42)**	**2.3×10^−9^**
**Saxena ** [**4**]	*CDKAL1*	rs10946398	1,684 (47.6)	1,300 (41.7)	1,513 (42.8)	1,426 (45.7)	341 (9.6)	393 (12.6)	**1.22 (1.12–1.33)**	**3.7×10^−6^**
	*IGFBP2*	rs4402960	1,633 (46.1)	1,339 (42.4)	1,558 (44)	1,441 (45.6)	352 (9.9)	382 (12.1)	**1.14 (1.05–1.24)**	**0.002**
**Steinthorsdottir ** [**5**]	*IGFBP2*	rs1470579	1,621 (45.7)	1,318 (41.7)	1,564 (44.1)	1,444 (45.6)	361 (10.2)	402 (12.7)	**1.17 (1.07–1.27)**	**0.0003**

Odds ratios were adjusted for age, gender and BMI under a multiplicative model

NGT: Normal Glucose Tolerant

T2D: Type 2 Diabetic

Allele 1: Major allele

Allele 2: Minor allele (tested)

### Replication studies in 4 additional independent populations

The 22 SNPs located in the 14 loci that we identified in our previous study were analyzed in other independent populations. For each polymorphism, the best fitting genetic model was selected from our previous whole-genome association study in French individuals [1]. Thus, all polymorphisms were analyzed under a multiplicative genetic model except for *EXT2* (dominant model) and *KCTD12* (recessive model). For *CDKAL1*, *CDKN2A/2B*, *IGFBP2*, *HHEX* and *EXT2*, we studied more than one polymorphism because they have all been reported as associated with T2D in previous studies [2]–[5].

We first analyzed another independent French group of more modest sample size (937 T2D and 1,000 NGT) (Table 2). Polymorphisms of predicted genes (*LOC646279*, OR_rs1256517_ = 1.44 [1.03–2.02], *P = *0.03; *LOC387761*, OR_rs7480010_ = 1.32 [1.01–1.72], *P = *0.04), *SLC30A8* (OR_rs13266634_ = 0.76 [0.59–0.97], *P* = 0.03), *MMP26* (OR_rs2499953_ = 2.45 [1.19–5.04], *P* = 0.01) and *CXCR4* (OR_rs932206 = _0.75 [0.57–0.97], *P* = 0.03) were more prevalent in T2D subjects than in NGT individuals. A trend towards T2D association was detected for one SNP of *EXT2* (OR_rs3740878_ = 0.50 [0.23–1.07], *P* = 0.07) and *HHEX* (OR_rs1111875 = _0.79 [0.60–1.02], *P* = 0.07).

**Table 2:** Genotypic distribution by number of individuals (%) of the 22 SNPs studied in the second set of French population

Whole-genome study	Gene	rs ID	Genotype 1-1		Genotype 1-2		Genotype 2-2		OR	*P*
			NGT	T2D	NGT	T2D	NGT	T2D	(95% CI)	
**Scott ** [**2**]	*CDKN2A/2B*	rs10811661	627 (66.3)	636 (69)	291 (30.8)	260 (28.2)	28 (3)	26 (2.8)	0.95 (0.70–1.30)	0.75
	*CDKN2A/2B*	rs564398	394 (41.9)	362 (40)	416 (44.2)	416 (46)	131 (13.9)	126 (13.9)	1.04 (0.81–1.32)	0.78
**Zeggini ** [**3**]	*CDKAL1*	rs7754840	399 (43.3)	368 (40.9)	426 (46.2)	414 (46)	96 (10.4)	118 (13.1)	1.10 (0.85–1.40)	0.47
	*CDKAL1*	rs7756992	469 (49.6)	419 (46.7)	398 (42.1)	378 (42.1)	78 (8.2)	100 (11.2)	1.22 (0.96–1.56)	0.11
**Saxena ** [**4**]	*CDKAL1*	rs10946398	413 (43.9)	372 (41.3)	432 (45.9)	410 (45.6)	96 (10.2)	118 (13.1)	1.09 (0.85–1.38)	0.5
	*IGFBP2*	rs4402960	434 (45.9)	404 (45.2)	420 (44.4)	387 (43.3)	91 (9.6)	103 (11.5)	1.17 (0.91–1.52)	0.22
**Steinthorsdottir ** [**5**]	*IGFBP2*	rs1470579	424 (45.2)	376 (45.4)	413 (44.1)	356 (43)	100 (10.7)	96 (11.6)	1.05 (0.81–1.35)	0.73
**Sladek ** [**1**]	*EXT2* d	rs1113132	540 (56.6)	502 (56.7)	359 (37.6)	324 (36.6)	55 (5.8)	60 (6.8)	0.82 (0.42–1.60)	0.57
	*EXT2* d	rs3740878	533 (56.5)	498 (61.5)	356 (37.7)	274 (33.8)	55 (5.8)	38 (4.7)	0.50 (0.23–1.07)	0.07
	*EXT2* d	rs11037909	528 (56.6)	409 (56.9)	348 (37.3)	264 (36.7)	56 (6)	46 (6.4)	0.71 (0.35–1.46)	0.35
	*EXT2* d	rs729287	533 (56.3)	499 (56.2)	359 (38)	331 (37.3)	54 (5.7)	58 (6.5)	0.71 (0.36–1.40)	0.32
	*HHEX*	rs1111875	334 (35.6)	279 (39)	465 (49.6)	337 (47.1)	139 (14.8)	99 (13.8)	0.79 (0.60–1.02)	0.07
	*HHEX*	rs7923837	479 (52.1)	474 (55)	369 (40.1)	330 (38.3)	72 (7.8)	58 (6.7)	0.81 (0.62–1.06)	0.12
	*LOC646279*	rs1256517	707 (74.8)	655 (74.1)	217 (23)	212 (24)	21 (2.2)	17 (1.9)	**1.44 (1.03**–**2.02)**	**0.03**
	*SLC30A8*	rs13266634	367 (38.5)	360 (43.5)	457 (48)	362 (43.7)	128 (13.4)	106 (12.8)	**0.76 (0.59**–**0.97)**	**0.03**
	*MMP26*	rs2499953	916 (96)	832 (93.2)	37 (3.9)	59 (6.6)	1 (0.1)	2 (0.2)	**2.45 (1.19**–**5.04)**	**0.01**
	*KCTD12* r	rs2876711	349 (36.9)	337 (37.8)	474 (50)	401 (45)	124 (13.1)	153 (17.2)	0.87 (0.62–1.23)	0.44
	*LDLR*	rs6413504	229 (24.1)	203 (24.9)	498 (52.5)	420 (51.4)	222 (23.4)	194 (23.8)	0.88 (0.69–1.13)	0.31
	*CAMTA1*	rs1193179	497 (53.6)	455 (55)	365 (39.3)	308 (37.2)	66 (7.1)	65 (7.8)	0.95 (0.73–1.26)	0.74
	*LOC387761*	rs7480010	484 (51.3)	324 (46.2)	376 (39.8)	293 (41.8)	84 (8.9)	84 (12)	**1.32 (1.01**–**1.72)**	**0.04**
	*NGN3*	rs10823406	552 (57.9)	534 (63.7)	350 (36.7)	277 (33)	51 (5.3)	27 (3.2)	0.79 (0.59–1.06)	0.12
	*CXCR4*	rs932206	312 (33.8)	257 (36.5)	433 (46.9)	340 (48.3)	178 (19.3)	107 (15.2)	**0.75 (0.57**–**0.97)**	**0.03**

Odds ratios were adjusted for age, gender and BMI under a multiplicative model except for *EXT2* (dominant) and *KCTD12* (recessive)

NGT: Normal Glucose Tolerant

T2D: Type 2 Diabetic

Allele 1: Major allele

Allele 2: Minor allele (tested)

d : Dominant genetic model

r : Recessive genetic model

Then, the same SNPs were analyzed in Austrians (504 T2D and 753 NGT) (Table 3). Associations between SNPs of *CDKN2A/2B* (OR_rs10811661 = _0.67 [0.52–0.87], *P* = 0.002), *IGFBP2* (OR_rs4402960 = _1.25 [1.02–1.53], *P* = 0.03), *SLC30A8* (OR_rs13266634 = _0.76 [0.61–0.94], *P* = 0.01) and *NGN3* (OR_rs7923837 = _0.73 [0.59–0.91], *P* = 0.005) loci and T2D were identified.

**Table 3 pone-0002031-t003:** Genotypic distribution by number of individuals (%) for the 22 SNPs studied in the Austrian population

Whole-genome study	Gene	rs ID	Genotype 1-1		Genotype 1-2		Genotype 2-2		OR	*P*
			NGT	T2D	NGT	T2D	NGT	T2D	(95% CI)	
**Scott ** [**2**]	*CDKN2A/2B*	rs10811661	455 (66.4)	328 (74)	202 (29.5)	105 (23.7)	28 (4.1)	10 (2.3)	**0.67 (0.52**–**0.87)**	**0.002**
	*CDKN2A/2B*	rs564398	235 (34.5)	160 (36.8)	328 (48.1)	190 (43.7)	119 (17.4)	85 (19.5)	0.93 (0.77–1.12)	0.44
**Zeggini ** [**3**]	*CDKAL1*	rs7754840	322 (47.5)	197 (48.4)	290 (42.8)	169 (41.5)	66 (9.7)	41 (10.1)	1.00 (0.81–1.24)	0.97
	*CDKAL1*	rs7756992	368 (53.1)	233 (51.8)	269 (38.8)	174 (38.7)	56 (8.1)	43 (9.6)	1.11 (0.91–1.37)	0.30
**Saxena ** [**4**]	*CDKAL1*	rs10946398	315 (48.2)	216 (48.9)	273 (41.7)	180 (40.7)	66 (10.1)	46 (10.4)	1.03 (0.84–1.27)	0.76
	*IGFBP2*	rs4402960	351 (53.1)	199 (45.5)	261 (39.5)	191 (43.7)	49 (7.4)	47 (10.8)	**1.25 (1.02**–**1.53)**	**0.03**
**Steinthorsdottir ** [**5**]	*IGFBP2*	rs1470579	328 (52.1)	209 (47.1)	245 (39)	185 (41.7)	56 (8.9)	50 (11.3)	1.17 (0.95–1.43)	0.14
**Sladek ** [**1**]	*EXT2* d	rs1113132	393 (57.6)	225 (51.6)	241 (35.3)	177 (40.6)	48 (7)	34 (7.8)	1.22 (0.73–2.02)	0.45
	*EXT2* d	rs3740878	399 (59.7)	230 (53.5)	223 (33.4)	169 (39.3)	46 (6.9)	31 (7.2)	1.15 (0.68–1.94)	0.61
	*EXT2* d	rs11037909	382 (59.5)	224 (53.6)	220 (34.3)	163 (39)	40 (6.2)	31 (7.4)	1.36 (0.79–2.33)	0.26
	*EXT2* d	rs729287	399 (59.5)	230 (52.6)	233 (34.7)	176 (40.3)	39 (5.8)	31 (7.1)	1.37 (0.79–2.37)	0.26
	*HHEX*	rs1111875	253 (37)	178 (41.3)	324 (47.4)	197 (45.7)	106 (15.5)	56 (13)	0.89 (0.73–1.07)	0.21
	*HHEX*	rs7923837	299 (43.8)	183 (41.8)	289 (42.4)	201 (45.9)	94 (13.8)	54 (12.3)	1.00 (0.82–1.22)	0.98
	*LOC646279*	rs1256517	553 (81.2)	343 (79)	119 (17.5)	85 (19.6)	9 (1.3)	6 (1.4)	1.15 (0.84–1.57)	0.40
	*SLC30A8*	rs13266634	331 (49)	240 (54.9)	283 (41.9)	167 (38.2)	62 (9.2)	30 (6.9)	**0.76 (0.61**–**0.94)**	**0.01**
	*MMP26*	rs2499953	657 (95.8)	431 (96.4)	29 (4.2)	16 (3.6)	0 (0)	0 (0)	0.81 (0.43–1.51)	0.50
	*KCTD12* r	rs2876711	260 (39.4)	155 (36.6)	301 (45.6)	185 (43.6)	99 (15)	84 (19.8)	1.17 (0.88–1.56)	0.27
	*LDLR*	rs6413504	172 (26.2)	93 (24)	334 (50.8)	207 (53.4)	151 (23)	88 (22.7)	1.06 (0.86–1.30)	0.58
	*CAMTA1*	rs1193179	377 (56.4)	223 (54.5)	249 (37.3)	157 (38.4)	42 (6.3)	29 (7.1)	1.04 (0.84–1.29)	0.69
	*LOC387761*	rs7480010	349 (52.2)	225 (51.7)	275 (41.2)	179 (41.1)	44 (6.6)	31 (7.1)	1.02 (0.82–1.27)	0.84
	*NGN3*	rs10823406	363 (54)	254 (59.1)	261 (38.8)	152 (35.3)	48 (7.1)	24 (5.6)	**0.73 (0.59**–**0.91)**	**0.005**
	*CXCR4*	rs932206	174 (26.7)	115 (28.8)	324 (49.7)	198 (49.5)	154 (23.6)	87 (21.8)	0.93 (0.77–1.13)	0.46

Odds ratios were adjusted for age, gender and BMI under a multiplicative model except for *EXT2* (dominant) and *KCTD12* (recessive)

NGT: Normal Glucose Tolerant

T2D: Type 2 Diabetic

Allele 1: Major allele

Allele 2: Minor allele (tested)

d : Dominant genetic model

r : Recessive genetic model

In Israeli Ashkenazi subjects (577 T2D and 552 NGT), *CDKN2A/2B* (OR_rs564398 = _1.26 [1.03–1.53], *P* = 0.02), *CDKAL1* (OR_rs7754840 = _1.30 [1.08–1.56], *P* = 0.005; OR_rs7756992 = _1.24 [1.03–1.49], *P* = 0.02), *IGFBP2* (OR_rs1470579 = _1.34 [1.12–1.61], *P* = 0.001) and *EXT2* (OR_rs729287 = _0.43 [0.21–0.88], *P* = 0.02) were more prevalent in the T2D group than in NGT individuals (Table 4).

**Table 4 pone-0002031-t004:** Genotypic distribution by number of individuals (%) of the 22 SNPs studied in the Israeli Ashkenazi population

Whole-genome study	Gene	rs ID	Genotype 1-1		Genotype 1-2		Genotype 2-2		OR	*P*
			NGT	T2D	NGT	T2D	NGT	T2D	(95% CI)	
**Scott ** [**2**]	*CDKN2A/2B*	rs10811661	371 (72.3)	395 (75)	130 (25.3)	122 (23.1)	12 (2.3)	10 (1.9)	0.89 (0.69–1.14)	0.34
	*CDKN2A/2B*	rs564398	271 (54.7)	251 (48.3)	190 (38.4)	220 (42.3)	34 (6.9)	49 (9.4)	**1.26 (1.03**–**1.53)**	**0.02**
**Zeggini ** [**3**]	*CDKAL1*	rs7754840	199 (43.4)	179 (34.9)	205 (44.8)	247 (48.1)	54 (11.8)	87 (17)	**1.30 (1.08**–**1.56)**	**0.005**
	*CDKAL1*	rs7756992	229 (48.2)	208 (40.5)	201 (42.3)	236 (46)	45 (9.5)	69 (13.5)	**1.24 (1.03**–**1.49)**	**0.02**
**Saxena ** [**4**]	*CDKAL1*	rs10946398	168 (39.2)	150 (35.1)	200 (46.6)	205 (48)	61 (14.2)	72 (16.9)	1.15 (0.95–1.39)	0.14
	*IGFBP2*	rs4402960	160 (35.3)	148 (30.3)	208 (45.9)	256 (52.5)	85 (18.8)	84 (17.2)	1.09 (0.90–1.31)	0.38
**Steinthorsdottir ** [**5**]	*IGFBP2*	rs1470579	191 (38.5)	155 (30.3)	232 (46.8)	252 (49.2)	73 (14.7)	105 (20.5)	**1.34 (1.12**–**1.61)**	**0.001**
**Sladek ** [**1**]	*EXT2* d	rs1113132	347 (65)	378 (70)	162 (30.3)	147 (27.2)	25 (4.7)	15 (2.8)	0.57 (0.29–1.12)	0.10
	*EXT2* d	rs3740878	343 (64.2)	369 (68.7)	165 (30.9)	151 (28.1)	26 (4.9)	17 (3.2)	0.65 (0.34–1.24)	0.19
	*EXT2* d	rs11037909	339 (64.8)	159 (30.4)	25 (4.8)	361 (69)	144 (27.5)	18 (3.4)	0.72 (0.38–1.35)	0.30
	*EXT2* d	rs729287	347 (65.7)	379 (70.8)	155 (29.4)	144 (26.9)	26 (4.9)	12 (2.2)	**0.43 (0.21**–**0.88)**	**0.02**
	*HHEX*	rs1111875	219 (40.9)	237 (44.1)	256 (47.9)	226 (42.1)	60 (11.2)	74 (13.8)	0.97 (0.81–1.16)	0.72
	*HHEX*	rs7923837	232 (43.2)	241 (44.7)	247 (46)	233 (43.2)	58 (10.8)	65 (12.1)	0.96 (0.80–1.16)	0.69
	*LOC646279*	rs1256517	403 (75.3)	393 (72.4)	122 (22.8)	135 (24.9)	10 (1.9)	15 (2.8)	1.18 (0.93–1.51)	0.18
	*SLC30A8*	rs13266634	278 (56.5)	301 (58.1)	183 (37.2)	187 (36.1)	31 (6.3)	30 (5.8)	0.97 (0.80–1.18)	0.76
	*MMP26*	rs2499953	481 (90.2)	505 (92.7)	52 (9.8)	38 (7)	0 (0)	2 (0.4)	0.77 (0.50–1.18)	0.22
	*KCTD12* r	rs2876711	295 (56.6)	272 (50.9)	189 (36.3)	229 (42.9)	37 (7.1)	33 (6.2)	1.24 (0.97–1.60)	0.09
	*LDLR*	rs6413504	145 (27.2)	134 (24.9)	257 (48.2)	278 (51.7)	131 (24.6)	126 (23.4)	1.01 (0.84–1.20)	0.95
	*CAMTA1*	rs1193179	297 (56)	310 (59.4)	198 (37.4)	179 (34.3)	35 (6.6)	33 (6.3)	0.92 (0.75–1.13)	0.42
	*LOC387761*	rs7480010	174 (33.1)	205 (38.2)	276 (52.5)	243 (45.2)	76 (14.4)	89 (16.6)	0.96 (0.80–1.15)	0.64
	*NGN3*	rs10823406	308 (59.8)	304 (56.7)	179 (34.8)	199 (37.1)	28 (5.4)	33 (6.2)	1.10 (0.89–1.35)	0.37
	*CXCR4*	rs932206	381 (71.2)	379 (69.8)	143 (26.7)	150 (27.6)	11 (2.1)	14 (2.6)	1.07 (0.84–1.36)	0.58

Odds ratios were adjusted for age, gender and BMI under a multiplicative model except for *EXT2* (dominant) and *KCTD12* (recessive)

NGT: Normal Glucose Tolerant

T2D: Type 2 Diabetic

Allele 1: Major allele

Allele 2: Minor allele (tested)

d : Dominant genetic model

r : Recessive genetic model

Finally, we also investigated a population of Moroccan origin (521 T2D and 423 NGT), in which only trends for association were found for SNPs of *CDKAL1* (OR_rs7754840 = _1.20 [0.99–1.46], *P* = 0.06; OR_rs10946398_ = 1.21 [0.99–1.47], *P* = 0.06) and *LOC646279* (OR_rs1256517 = _0.84 [0.69–1.02], *P* = 0.07) (Table 5).

**Table 5:** Genotypic distribution by number of individuals (%) of the 22 SNPs studied in the Morrocan population

Whole-genome study	Gene	rs ID	Genotype 1-1		Genotype 1-2		Genotype 2-2		OR	*P*
			NGT	T2D	NGT	T2D	NGT	T2D	(95% CI)	
**Scott ** [**2**]	*CDKN2A/2B*	rs10811661	288 (69.4)	368 (71)	116 (27.9)	127 (24.5)	11 (2.6)	23 (4.4)	0.99 (0.78–1.26)	0.97
	*CDKN2A/2B*	rs564398	243 (58)	323 (62.6)	148 (35.3)	170 (33)	28 (6.7)	23 (4.5)	0.83 (0.66–1.03)	0.09
**Zeggini ** [**3**]	*CDKAL1*	rs7754840	204 (48.8)	232 (44.8)	176 (42.1)	219 (42.3)	38 (9.1)	67 (12.9)	1.20 (0.99–1.46)	0.06
	*CDKAL1*	rs7756992	204 (48.9)	238 (45.8)	172 (41.2)	225 (43.3)	41 (9.8)	57 (11)	1.12 (0.92–1.37)	0.25
**Saxena ** [**4**]	*CDKAL1*	rs10946398	204 (48.8)	231 (44.6)	176 (42.1)	220 (42.5)	38 (9.1)	67 (12.9)	1.21 (0.99–1.47)	0.06
	*IGFBP2*	rs4402960	149 (36.3)	169 (32.6)	196 (47.7)	253 (48.7)	66 (16.1)	97 (18.7)	1.11 (0.92–1.33)	0.28
**Steinthorsdottir ** [**5**]	*IGFBP2*	rs1470579	133 (31.7)	145 (28.1)	199 (47.4)	247 (47.8)	88 (20.9)	125 (24.2)	1.12 (0.94–1.34)	0.22
**Sladek ** [**1**]	*EXT2* d	rs1113132	317 (75.7)	395 (76.1)	97 (23.1)	114 (22)	5 (1.2)	10 (1.9)	1.55 (0.52–4.64)	0.43
	*EXT2* d	rs3740878	306 (72.7)	388 (75)	110 (26.1)	119 (23)	5 (1.2)	10 (1.9)	1.53 (0.51–4.58)	0.45
	*EXT2* d	rs11037909	308 (73.7)	386 (74.8)	104 (24.9)	120 (23.3)	6 (1.4)	10 (1.9)	1.30 (0.46–3.64)	0.84
	*EXT2* d	rs729287	319 (75.8)	397 (76.3)	97 (23)	114 (21.9)	5 (1.2)	9 (1.7)	1.44 (0.47–4.40)	0.52
	*HHEX*	rs1111875	177 (42.2)	236 (46)	187 (44.6)	227 (44.2)	55 (13.1)	50 (9.8)	0.87 (0.71–1.05)	0.15
	*HHEX*	rs7923837	227 (53.8)	300 (57.9)	158 (37.4)	183 (35.3)	37 (8.8)	35 (6.8)	0.88 (0.72–1.08)	0.21
	*LOC646279*	rs1256517	182 (43.3)	248 (48.5)	188 (44.8)	216 (42.3)	50 (11.9)	47 (9.2)	0.84 (0.69–1.02)	0.07
	*SLC30A8*	rs13266634	292 (69.4)	360 (69.6)	118 (28)	145 (28.1)	11 (2.6)	12 (2.3)	0.99 (0.77–1.27)	0.95
	*MMP26*	rs2499953	372 (89.9)	451 (88.6)	40 (9.7)	57 (11.2)	2 (0.5)	1 (0.2)	1.06 (0.71–1.59)	0.78
	*KCTD12* r	rs2876711	192 (48.9)	271 (52.1)	164 (41.7)	204 (39.2)	37 (9.4)	45 (8.7)	0.89 (0.68–1.16)	0.37
	*LDLR*	rs6413504	139 (33.4)	180 (34.8)	208 (50)	242 (46.7)	69 (16.6)	96 (18.5)	0.98 (0.82–1.18)	0.85
	*CAMTA1*	rs1193179	134 (31.9)	150 (29.4)	213 (50.7)	261 (51.1)	73 (17.4)	100 (19.6)	1.09 (0.90–1.32)	0.36
	*LOC387761*	rs7480010	151 (36.4)	173 (33.3)	201 (48.4)	249 (48)	63 (15.2)	97 (18.7)	1.11 (0.92–1.34)	0.26
	*NGN3*	rs10823406	304 (72.9)	351 (69.2)	100 (24)	142 (28)	13 (3.1)	14 (2.8)	1.16 (0.91–1.49)	0.24
	*CXCR4*	rs932206	305 (72.5)	346 (67.2)	102 (24.2)	153 (29.7)	14 (3.3)	16 (3.1)	1.18 (0.92–1.51)	0.19

Odds ratios were adjusted for age, gender and BMI under a multiplicative model except for *EXT2* (dominant) and *KCTD12* (recessive)

NGT: Normal Glucose Tolerant

T2D: Type 2 Diabetic

Allele 1: Major allele

Allele 2: Minor allele (tested)

d : Dominant genetic model

r : Recessive genetic model

### Gene-gene interactions and cumulative genetic effects in French subjects

For each studied locus, we selected the most T2D-associated SNP, including *TCF7L2* rs7903146 (N = 15) in order to assess their cumulative effects on T2D prevalence as well as potential gene-gene interactions (i.e., deviation from a multiplicative model) in the complete set of French individuals (4,232 T2D and 4,595 NGT) [6].

All pairwise combinations (N = 253) were studied for the 15 SNPs (Figure S1). We found interactions of *IGFBP2* rs1470579 with *LOC387761* rs7480010 (*P* = 0.04), *SLC30A8* rs13266634 (*P* = 0.007) and *HHEX* rs7923837 (*P* = 0.01). Similarly, we detected interactions of *IGFBP2* rs4402960 with the same loci (*P* = 0.02, *P* = 0.01 and *P* = 0.008, respectively). Furthermore, interactions of *CXCR4* rs932206 with *NGN3* rs10823406 (*P* = 0.02) and *CDKN2A/2B* rs10811661 (*P* = 0.02) were also found. No antagonistic effects were detected.

**Figure 1:**
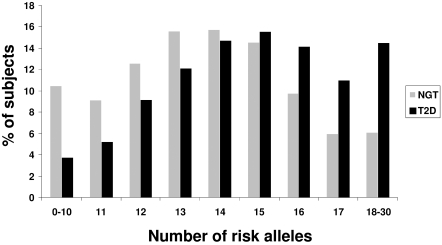
Percentage distribution of NGT and T2D individuals by number of risk alleles.

**Figure 2:**
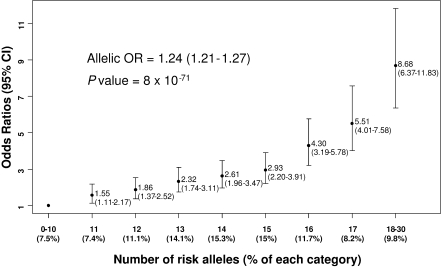
Odds ratios for French individuals carrying increasing numbers of risk alleles. Fifteen T2D genetic variants identified by GWA studies were analyzed in 4,232 T2D and 4,595 NGT French subjects for their cumulative effects on T2D prevalence. Allelic ORs and 95%-CIs were calculated using logistic regression model adjusted for age, gender and BMI. They are presented for each group, defined by their number of T2D risk alleles. Each additional allele increases in average the risk to develop T2D by 24 % (*P* = 8×10^−71^).

The percentages of individuals with increasing numbers of risk alleles in T2D subjects and NGT individuals are shown in Figure 1. For all SNPs, including those of *EXT2* and *KCTD12*, we considered their multiplicative allelic effects on T2D prevalence. The ORs for T2D in subjects carrying increasing numbers of risk alleles are presented in Figure 2 in comparison to the 7.5% of the study population in the reference group that have 0 to 10 risk alleles. After adjustment for age, body mass index (BMI) and gender, each additional risk allele increased the odds of disease by 1.24 [1.21–1.27]. Individuals with at least 18 risk alleles (6.1% of NGT individuals and 14.5% of T2D subjects) had an OR of 8.68 [6.37–11.83] compared to the reference group.

**Figure 3:**
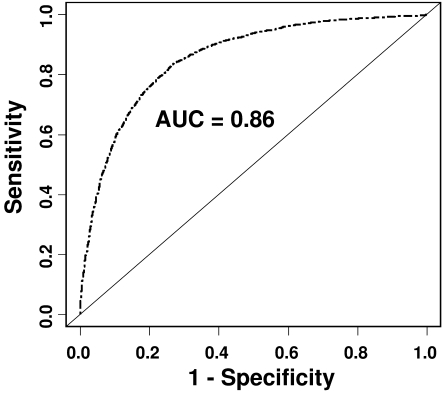
ROC for the information provided by the 15 T2D-associated genetic variants. We used a logistic regression model adjusted for age, gender and BMI including a covariate for the number of risk alleles. The area under the ROC curve was 0.86. Academic point system: 0.90–1 = excellent; 0.80–0.90 = good; 0.70–0.80 = fair; 0.60–0.70 = poor; 0.50–0.60 = fail.

The predictive power of tests can be evaluated by the area under the ROC curve (AUC) [7]. The AUC is a measure of the discriminatory power of the test. A perfect test would have an AUC of 1; a test with no discriminatory power, an AUC of 0.5. For the 15 polymorphisms, the area under the ROC curve is 0.86 (Figure 3), corresponding to a high predictive power.

## Discussion

GWA scans in Europeans [1]–[5] and other populations [8]–[11] have recently identified and confirmed new genetic variants associated with T2D. However, the clinical interest of these discoveries will depend on their true contribution in other ethnic groups as well as on their predictive value for T2D risk.

Using the same study design as our French GWA scan (joint stage I and fast track stage II), we confirmed the strong association of *CDKAL1*, *CKN2A/2B* and *IGFBP2* SNPs with T2D previously found by other GWA scans [2]–[5]. Intriguingly, these results were not replicated in another independent French population. This may be due to the modest sample size and/or because T2D cases and NGT controls were not specifically selected from families with or without history of diabetes, respectively, and/or because T2D cases and NGT controls were not matched for BMI. Our data show that the level of association of some SNPs with T2D depends on factors other than just ethnicity. Similar discordant results were also found among European cohorts from Finland [2]. However, we replicated our previous results for *SLC30A8*, *MMP26*, *CXCR4*, *LOC387761* and *LOC646279* SNPs, suggesting that these loci may be truly involved in T2D risk in the French population.

In another mid-sized population from Central Europe (Austria), associations with T2D were found only with *CDKN2A/2B*, *IGFBP2*, *SLC30A8*, and *NGN3* SNPs supporting the value of using large homogeneous populations to characterize the T2D genetic architecture in a given population. In Israeli Ashkenazi individuals, in whom one *EXT2* SNP was found to be a T2D genetic marker, *CDKAL1*, *CDKN2A/2B* and *IGFBP2* SNPs were associated with T2D, as in French subjects. Even though the *TCF7L2* rs790146 SNP was previously associated with T2D in this Moroccan group [12], no association was detected with any of the newly studied SNPs. While this lack of association may be due to the modest sample size or hidden biases such as population stratification, it may also signify that other SNPs in the same genes or located in different genes could be associated in the North African population.

Among the 14 studied loci, only 3 (*CDKN2A/2B*, *IGFBP2* and *SLC30A8*) were found to be associated with T2D in at least 3 independent case-control groups (Table 6). Their association was recently confirmed in other subjects of European or Japanese origin, as for *CDKAL1* and *HHEX* [13]–[17]. For the other loci, further meta-analyses and systematic replications of GWA studies (phase 2) are now necessary to fully evaluate their contribution to T2D risk. In the light of discrepancies between studied populations, GWA scans in non-European ethnic groups may bring additional insight. Further efforts in re-sequencing [18] are also needed to find etiologic variants causing T2D.

**Table 6:** Summary of replication results in the 5 case-control groups

Whole-genome study	Gene	French (First set)	French (Second set)	Austrian	Israeli Ashkenazi	Morrocan	Total
**Scott ** [**2**]	*CDKN2A/2B*	+	-	+	+	-	**+++**
**Zeggini ** [**3**]	*CDKAL1*	+	-	-	+	-	++
**Saxena ** [**4**]	*IGFBP2*	+	-	+	+	-	**+++**
**Steinthorsdottir ** [**5**]							
**Sladek ** [**1**]	*EXT2*	(+)	-	-	+	-	++
	*HHEX*	(+)	-	-	-	-	+
	*LOC646279*	(+)	+	-	-	-	++
	*SLC30A8*	(+)	+	+	-	-	**+++**
	*MMP26*	(+)	+	-	-	-	++
	*KCTD12*	(+)	-	-	-	-	+
	*LDLR*	(+)	-	-	-	-	+
	*CAMTA1*	(+)	-	-	-	-	+
	*LOC387761*	(+)	+	-	-	-	++
	*NGN3*	(+)	-	+	-	-	++
	*CXCR4*	(+)	+	-	-	-	++

+: At least one SNP detected associated with T2D

-: No SNP detected associated with T2D

( ) : Previously known data

There is a growing interest in analyzing the combined effect of these novel loci on T2D susceptibility. This is the first time, to our knowledge, that potential synergistic interactions between novel T2D loci have been shown. *IGFBP2* SNPs seem to interact with *LOC38776*, *SLC30A8* and *HHEX* genetic variants. These data are in agreement with a primary beta-cell dysfunction. IGF2BP2 binds to the key growth and insulin signaling molecule insulin-like growth factor 2 (IGFII) and is highly expressed in pancreatic islets [3]. The zinc transporter ZnT8 (*SLC30A8* protein) is specifically expressed in pancreatic endocrine cells and may participate in regulating insulin exocytosis [19], [20]. Similarly, the *CXCR4* risk variant was found to interact with *NGN3* and *CDKN2A/2B* SNPs. It was reported that CXCR4-positive pancreatic cells express markers of pancreatic endocrine progenitors such as *NGN3* [21]. *CDKN2A* could be a possible biological candidate for T2D [22] as its over-expression in rodents causes a decrease in islet proliferation [23]. The emerging picture of these possible interactions emphasizes the ability of multiple SNPs to potentiate their deleterious effects in both beta-cell development and function.

The evaluation of the T2D risk in individuals carrying increasing numbers of risk variants is critical for a potential clinical use of a genetic test in the general population. It was previously shown that UK individuals with *TCF7L2*, *PPARG* and *KCNJ11* risk alleles had an OR of 5.71-fold (95% CI, 1.15 to 28.3) compared to those with no risk alleles, for an AUC of 0.58 [6]. It has been suggested that 20–25 risk variants with allele frequencies greater than 0.1 and ORs of 1.5 are required for an AUC of about 0.8 [24]. In our study, with only 15 SNPs, we reached a good discriminating power to identify individuals with high susceptibility for T2D. After adjustments for age, BMI and gender, subjects with at least 18 risk alleles (14.5% of French T2D subjects) had approximately 9-fold higher risk of developing T2D compared to the reference group, for an AUC of 0.86. If confirmed, this increase in T2D risk due to genetic factors is even higher than that due to severe obesity (OR = 7.37) [25], the most established T2D risk factor.

After many years of limited success, the genetic architecture of T2D (common SNPs) is finally being uncovered by GWA studies. Even though the absence or presence of association in a given population is dependent on many factors (e.g., number of individuals, ethnicity, SNP prevalence, BMI, familial history of T2D and others), replication studies certainly help in validating SNPs truly associated with T2D and in excluding false positives.

Our data support the concept that T2D loci may interact together. Consequently, while single polygenic susceptibility variants may be of limited use in disease prediction, the combined information from a number of these variants should allow the identification of groups of subjects at high and low risk of developing a complex disease [26]. Hence, our study opens up the way to new applications in public health, based on early genetic testing for better prevention and care.

## Materials and Methods

### Study design

We first investigated the association with T2D of *CDKAL1*, *CDKN2A/2B* and *IGFBP2* SNPs in the same French population analyzed in our previous GWA study (3,295 T2D and 3,595 NGT). These 3 loci were selected for their high degree of association with T2D in GWA studies and their ability to be replicated in different populations of European origin [2]–[5]. In addition to these 3 loci, the role of 11 additional loci for which we found the 15 highest SNP association signals (except for *TCF7L2*) in the joint stage I and fast track stage II of the French GWA scan [1] (*SLC30A8, HHEX, EXT2, LOC646279, MMP26, KCTD12, LDLR, CAMTA1, LOC38776, NGN3* and *CXCR4*) was evaluated in 4 additional independent groups (2,539 T2D and 2,728 NGT) of European (French, Austrian and Israeli Ashkenazi) and non-European (Moroccan) origin. Finally, in all French subjects, we assessed the cumulative genetic risk of carrying the studied risk alleles on T2D prevalence and their possible interactions, including the previously discovered T2D-associated *TCF7L2* rs7903146 SNP.

### Study populations

The main clinical characteristics of each studied population were presented in Table S1. The first French group of T2D subjects and NGT controls was previously described [1].The second set of French samples includes NGT individuals from the “Supplémentation en Vitamines et Minéraux Antioxydants” (SU.VI.MAX) cohort [27] and T2D subjects from the “DIABete de type 2, NEPHROpathie et GENEtique” (DIAB2.NEPHRO.GENE) study [28].

The SU.VI.MAX study was a French population-based prevention trial designed to evaluate the impact of a daily antioxidant supplementation at nutritional doses on the incidence of ischemic heart disease and cancer. All participants were recruited from throughout France between October 1994 and June 1995. [29]. The NGT controls were selected when having no hypoglycemic treatment and a fasting glucose <6.1 mmol/l. At baseline, a 35-mL venous blood sample was obtained from participants who had been fasting for 12 hr at the time of the visit. The samples were collected in vacutainer tubes that do not interfere with the concentration of trace elements (Becton Dickinson). After collection, blood was kept at +4°C in the dark until centrifugation. Centrifugation was realized at standard time, gravity, and temperature. The time elapsing between collection and aliquoting was recorded for all samples; it was less than 1 hr. Aliquots were stored at −20°C in the mobile units and field centers for, at the most, 7 days prior to shipment in dry ice to the reference laboratories and coordinating center.

The DIAB2.NEPHRO.GENE study was a French multi-center case-control study (15 diabetes and 5 nephrology centers from throughout France between 2001 and 2004) designed to assess the genetic determinants of diabetic nephropathy in type 2 diabetes. T2D was diagnosed on clinically determined absence of type 1 or secondary diabetes, in individuals of more than 40 years of age at onset and without insulin treatment within 2 years after disease onset. Each patient record was carefully checked by an adjudication committee to ascertain T2D status and diabetic complications. 21 ml of blood drawn on EDTA were used for DNA extraction using standard procedures (ethanol precipitation) and samples were stored at –80°c until use. The number of recruited people was 11 in December 2001 (first month of the study), 491 subjects in 2002, 514 in 2003, 593 in 2004, 897 in 2005 and 229 thereafter.

The Austrian subjects were of Bavarian and Austrian German descent and came from the greater region of Salzburg, Austria. They were recruited between April 1999 and December 2002. Unrelated patients with type 2 diabetes were recruited from diabetes outpatient clinics of the Landeskliniken Salzburg and the Hospital Hallein (near Salzburg). The diagnosis of T2D was based on use of hypoglycemic agents or plasma glucose values >126 mg/dl (in absence of treatment). The patients were seen repeatedly and managed by the outpatient clinics. Participants in the Salzburg Atherosclerosis Prevention Program in Subjects at High Individual Risk (SAPHIR) [12], [30]–[32] who were not using hypoglycemic medications and had fasting plasma glucose levels <110 mg/dL served as NGTcontrols. SAPHIR was a population-based prospective study that investigated the involvement of factors contributing to the control of plasma lipid transport and carbohydrate metabolism in the progression of atherosclerotic vascular disease. Unrelated men and women subjects with an age range between 39 and 67 years who live in the greater Salzburg region and responded to invitations by their family or workplace physician or to announcement in the local press were included in the study. Average rates of recruitment were 10 controls/week and 3 T2D subjects/week. For all samples, whole blood was collected after an overnight fast in tubes containing 1.6 mg/ml EDTA. Plasma was separated <30 min after collection and used immediately for analysis. Aliquots were stored at –70°C. Aliquots of blood collected in EDTA were stored at –70°C. Genomic DNA was extracted from whole blood and stored at –20°C prior to analysis.

All Israeli NGT/T2D subjects lived and were ascertained in Israel with the help of 15 major diabetes treatment centers throughout the country. They were of Ashkenazi Jewish origin, defined as having all 4 grandparents born in Northern or Eastern Europe. Subjects with known or suspected Sephardic Jewish or non-Jewish ancestry were excluded. Thus, only Jewish subjects were recruited and Jewish patients from the Mediterranean basin, the Persian Gulf region (Iraq, Iran), Yemen, Ethiopia and other areas that were populated with Jews before the major Roman exile of 70 C.E. were not included. The T2D patients were ascertained between 2002 and 2004 in 15 diabetes clinics throughout Israel by the “Israel Diabetes Research Group” and were defined according to WHO criteria (fasting glucose >140mg/dl on two or more occasions, or random glucose >200 mg/dl on two or more occasions). To avoid late-onset type 1 diabetics, patients who became insulin-dependent within 2 years of diagnosis were excluded. The average age at diagnosis was 47 years old. The NGT controls were defined as Ashkenazi (Northern and Eastern European ancestry) with no history of glucose intolerance or T2D and were purchased from the National Laboratory for the Genetics of Israeli Populations (http://www.tau.ac.il/medicine/NLGIP/nlgip.htm). They denied ever having been diagnosed with elevated blood glucose level, T2D or glucose intolerance. Whole blood samples were obtained in vacuum tubes containing EDTA. The samples were stored at 4°C and transferred to the Endocrine Laboratory at the Hadassah Hospital, Jerusalem Israel within 72h of collection. DNA was extracted using the Puregene Genomic DNA extraction kit purchased from Gentra Systems, Minneapolis, MN, U.S.A. according to the manufacturer's recommendations. Concentrated DNA was stored at −80°C, diluted stocks were stored at −20°C and working solutions were stored at 4°C.

Moroccan subjects were recruited, between February and July 2006, by the Faculty of Medicine (Fes) within the framework of the Genetic project Diabetes Morocco (GenDiabM: ∼180 subjects by month) and were subjected to a standardized clinical examination at the Hassan II Hospital and in regional health centers. The NGT and T2D subjects were from two Moroccan regions: Fes-Tounate (central-North region) and Rabat-Sale (western region). Patients with T2D were recruited from a registry of associations for T2D and health centers when they had a family history of T2D in first degree relatives. The diagnosis of T2D was made according to the 1997 American Diabetes Association criteria or on being treated with medications for diabetes. The NGT controls were recruited from an unselected population undergoing a routine health check-up at the same health centers. All control individuals ≥40 years of age, not previously diagnosed for T2D, with no history of T2D in first-degree relatives, and with fasting plasma glucose <6.1 mmol/l. Blood and serum samples (9 ml) were collected from all individuals and were immediately stored in frozen conditions (serum at −20°C and blood at −80°C) until use. Genomic DNA was then extracted from whole blood and stored at −20°C.

In all populations, the studied individuals were unrelated to each other (with no first-degree relatives). This genetic study was approved by local Ethical Committees and written informed consent was obtained from all participants.

### SNP genotyping

All the polymorphisms were genotyped using an allelic discrimination assay-by-design TaqMan method on ABI 7900 (Applied Biosystems). All genotypic distributions were in Hardy-Weinberg equilibrium. The genotyping success rate was higher than 98% for each SNP. For each SNP, the genotyping error rate was reported in Table S2 and assessed by randomly re-genotyping 384 participants in each population.

### Statistical analysis

Odds ratios were assessed by logistic regression models adjusted for age, BMI and gender. In Tables 2–5 and in Table S2, we tested the effect of the minor allele. In these tables, the risk allele is the minor allele if the OR is >1 and is the major allele if the OR is <1. The replication of an association with T2D was considered positive on the condition that the risk allele was not different from what was found in previous GWA studies. For each population, a simple Bonferroni correction (multiplication by the number of SNPs) was applied to the *P* values for multiple comparisons. After correction, no association remained significant, except for those found in our original French population. However, in the context of replication, it remains unlikely to detect an effect due to statistical fluctuation only. We explored the effect of multiple SNPs using a logistic regression model including a variable for the number of risk alleles in order to quantify the risk per supplementary allele for all the variants included in the model. OR corresponding to a given number of risk alleles compared to the reference group was also calculated. We evaluated the ability of this model to discriminate between NGT and T2D individuals with a Receiver Operating Characteristic (ROC) curve [33] using a logistic regression model adjusted for age, gender and BMI including a covariate for the number of risk alleles of the 15 studied variants. The Area Under the ROC Curve (AUC) was calculated as a measure of the discriminative power of the test. Gene-gene interactions (i.e., deviation from a multiplicative model) were tested by comparing a logistic regression model including only the main effects to another model including the main effects and an interaction term with a likelihood ratio test. We further explored the way two variants interact with interaction plots. We used Quanto (http://hydra.usc.edu/GxE/) for power calculations. Pairwise linkage disequilibrium between genetic markers was assessed using the R “genetics” package (version 1.3.2). All *P* values are two-sided. SPSS (version 14.0.2) and R statistics (version 2.5.1) software were used for general statistical analysis.

## Supporting Information

Figure S1 Significant gene-gene interactions between genetic variants and T2D. 1: Homozygous for the protective allele; 2: Heterozygous; 3: Homozygous for the risk allele. The minimum number of samples by genotype intersection and by status is presented thereafter. A: 48, B: 27, C: 43, D: 37, E: 41, F: 31, G: 42, H: 16.
(0.24 MB TIF)

Table S1 Clinical characteristics of the studied populations.
(0.04 MB DOC)

Table S2 Linkage disequilibrium and minimum detectable effect size with a statistical power of 80% for the 22 SNPs in the 5 case-control groups.
(0.12 MB DOC)
