# Supplementary material for: Post Genome-Wide Association Studies of Novel Genes Associated with Type 2 Diabetes Show Gene-Gene Interaction and High Predictive Value
Source: PLoS One. 2008 May 7;3(5):e2031. doi: 10.1371/journal.pone.0002031 (PMC2346547; doi:10.1371/journal.pone.0002031)
Supplement: Table S1 — Clinical characteristics of the studied populations. (0.04 MB DOC) [file pone.0002031.s002.doc]

**Table S1.**

**Clinical characteristics of the studied populations**

| **Variable** | **French (first set)** | | **French (second set)** | | **Austrian** | | **Morrocan** | | **Israeli Ashkenazi** | |
| --- | --- | --- | --- | --- | --- | --- | --- | --- | --- | --- |
|  | **T2D** | **NGT** | **T2D** | **NGT** | **T2D** | **NGT** | **T2D** | **NGT** | **T2D** | **NGT** |
| **N** | 3,295 | 3,595 | 937 | 1,000 | 504 | 753 | 521 | 423 | 577 | 552 |
| **Sex ratio (men / women)** | 2,035 / 1,260 | 1,521 / 2,074 | 578 / 359 | 428 / 572 | 298 / 206 | 462 / 291 | 159 / 362 | 132 / 291 | 279 / 298 | 209 / 343 |
| **Age (years)** | 62 ± 11 | 56 ± 10 | 66 ± 10 | 50 ± 6 | 57 ± 10 | 52 ± 6 | 58 ± 11 | 55 ± 12 | 63 ± 10 | 56 ± 26 |
| **BMI (kg/m²)** | 28.34 ± 3.66 | 24.87 ± 3.31 | 31.14 ± 5.66 | 24.07 ± 3.47 | 30.50 ± 6.37 | 26.77 ± 4.00 | 28.01 ± 4.75 | 27.23 ± 5.30 | 28.97 ± 4.96 | 26 ± 3.99 |

Data are presented as means ± standard deviation.

T2D: Type 2 Diabetic

NGT: Normal Glucose Tolerant
