# Supplementary material for: Post Genome-Wide Association Studies of Novel Genes Associated with Type 2 Diabetes Show Gene-Gene Interaction and High Predictive Value
Source: PLoS One. 2008 May 7;3(5):e2031. doi: 10.1371/journal.pone.0002031 (PMC2346547; doi:10.1371/journal.pone.0002031)
Supplement: Table S2 — Linkage disequilibrium and minimum detectable effect size with a statistical power of 80% for the 22 SNPs in the 5 case-control groups. (0.12 MB DOC) [file pone.0002031.s003.doc]

**Table S2.**

**Linkage disequilibrium and minimum detectable effect size with a statistical power of 80% for the 22 SNPs in the 5 case-control groups**

| **Gene** | **Alleles** | **rs ID** | **French (first set)** | | | **French (second set)** | | | **Austrian** | | | **Morrocan** | | | **Israeli Ashkenazi** | | |
| --- | --- | --- | --- | --- | --- | --- | --- | --- | --- | --- | --- | --- | --- | --- | --- | --- | --- |
|  | **1-2** |  | **Error** | **r²** | **Effect** | **Error** | **r²** | **Effect** | **Error rate** | **r²** | **Effect** | **Error** | **r²** | **Effect** | **Error** | **r²** | **Effect** |
|  |  |  | **rate** |  | **size** | **rate** |  | **size** |  |  | **size** | **rate** |  | **size** | **rate** |  | **size** |
| *CDKN2A/2B* | A/G | rs564398 | 0 | 0 | 0.91 | 0 | 0 | 0.83 | 0 | 0 | 0.79 | 0 | 0 | 0.73 | 0 | 0 | 0.77 |
| *CDKN2A/2B* | C/T | rs10811661 | 0 |  | 0.88 | 0 |  | 0.78 | 0.005 |  | 0.72 | 0 |  | 0.71 | 0.003 |  | 0.71 |
| *CDKAL1* | A/C | rs10946398 (a) | 0 | a-b: 0.73 | 1.10 | 0 | a-b: 0.99 | 1.21 | 0 | a-b: 0.99 | 1.28 | 0 | a-b: 1.00 | 1.31 | 0 | a-b: 0.60 | 1.27 |
| *CDKAL1* | C/G | rs7754840 (b) | 0 | a-c: 0.99 | 1.10 | 0 | a-c: 0.70 | 1.21 | 0 | a-c: 0.67 | 1.28 | 0 | a-c: 0.61 | 1.31 | 0 | a-c: 0.30 | 1.27 |
| *CDKAL1* | A/G | rs7756992 (c) | 0.005 | b-c: 0.73 | 1.11 | 0.008 | b-c: 0.70 | 1.21 | 0 | b-c: 0.70 | 1.28 | 0.005 | b-c: 0.61 | 1.31 | 0 | b-c: 0.52 | 1.28 |
| *IGFBP2* | G/T | rs4402960 | 0.003 | 0.97 | 1.10 | 0 | 0.97 | 1.21 | 0 | 0.92 | 1.28 | 0.003 | 0.83 | 1.30 | 0 | 0.76 | 1.27 |
| *IGFBP2* | A/C | rs1470579 | 0 |  | 1.10 | 0 |  | 1.21 | 0 |  | 1.28 | 0 |  | 1.30 | 0 |  | 1.27 |
| *EXT2^d^* | C/G | rs1113132 (a) | na | a-b: na | na | 0 | a-b: 0.99 | 0.55 | 0.003 | a-b: 0.97 | 0.45 | 0 | a-b: 0.91 | 0.15 | 0 | a-b: 0.98 | 0.34 |
| *EXT2^d^* | C/T | rs11037909 (b) | na | b-c: na | na | 0 | b-c: 0.97 | 0.55 | 0 | b-c: 0.96 | 0.45 | 0 | b-c: 0.95 | 0.15 | 0 | b-c: 0.99 | 0.34 |
| *EXT2 ^d^* | A/G | rs3740878 (c) | na | a-c: na | na | 0.005 | a-c: 0.96 | 0.55 | 0 | a-c: 0.92 | 0.45 | 0 | a-c: 0.90 | 0.15 | 0.008 | a-c: 0.98 | 0.34 |
| *EXT2 ^d^* | C/T | rs729287 (d) | na | b-d: na | na | 0 | b-d: 0.98 | 0.55 | 0 | b-d: 0.93 | 0.45 | 0 | b-d: 0.89 | 0.15 | 0 | b-d: 0.91 | 0.34 |
|  |  |  |  | a-d: na |  |  | a-d: 0.98 |  |  | a-d: 0.91 |  |  | a-d: 0.97 |  |  | a-d: 0.93 |  |
|  |  |  |  | c-d: na |  |  | c-d: 0.95 |  |  | c-d: 0.91 |  |  | c-d: 0.88 |  |  | c-d: 0.91 |  |
| *HHEX* | A/G | rs1111875 | na | na | na | 0 | 0.77 | 0.83 | 0 | 0.71 | 0.79 | 0 | 0.61 | 0.76 | 0 | 0.76 | 0.78 |
| *HHEX* | A/G | rs7923837 | na | na | na | 0 |  | 0.81 | 0 |  | 0.78 | 0 |  | 0.73 | 0 |  | 0.78 |
| *LOC646279* | C/T | rs1256517 | na | na | na | 0 | na | 1.29 | 0.005 | na | 1.43 | 0.005 | na | 1.32 | 0.008 | na | 1.37 |
| *SLC30A8* | C/T | rs13266634 | na | na | na | 0 | na | 0.83 | 0 | na | 0.78 | 0 | na | 0.68 | 0 | na | 0.75 |
| *MMP26* | A/G | rs2499953 | na | na | na | 0 | na | 1.62 | 0 | na | 2.01 | 0 | na | 1.72 | 0 | na | 1.64 |
| *KCTD12^r^* | C/T | rs2876711 | na | na | na | 0.005 | na | 0.77 | 0 | na | 0.72 | 0.003 | na | 0.69 | 0 | na | 0.71 |
| *LDLR* | A/G | rs6413504 | na | na | na | 0 | na | 0.83 | 0 | na | 0.79 | 0 | na | 0.76 | 0 | na | 0.79 |
| *CAMTA1* | A/G | rs1193179 | na | na | na | 0.003 | na | 1.22 | 0.005 | na | 1.30 | 0 | na | 1.30 | 0.003 | na | 1.30 |
| *LOC387761* | A/G | rs7480010 | na | na | na | 0 | na | 1.22 | 0 | na | 1.28 | 0 | na | 1.31 | 0 | na | 1.27 |
| *NGN3* | A/G | rs10823406 | na | na | na | 0 | na | 0.81 | 0 | na | 0.76 | 0 | na | 0.68 | 0 | na | 0.75 |
| *CXCR4* | A/G | rs932206 | na | na | na | 0 | na | 0.83 | 0 | na | 0.79 | 0.003 | na | 0.68 | 0 | na | 0.71 |

Allele 1: Major allele

Allele 2: Minor allele (tested)

r²: Linkage Disequilibrium

*^d^*: Dominant genetic model

*^r^*: Recessive genetic model

Error rate: assessed by re-genotyping 384 random samples

na: Not applicable
